# MiR-3150b inhibits hepatocellular carcinoma cell proliferation, migration and invasion by targeting GOLPH3

**DOI:** 10.1136/jim-2019-001181

**Published:** 2019-12-05

**Authors:** Yi Zhang, Jianjun Wang, Hongling Su

**Affiliations:** 1 Department of General Surgery, Xi’an XD Group Hospital, Xi'an, China; 2 Department of Gastroenterology, Xi'an XD Group Hospital, Xi'an, China

**Keywords:** Golgi phosphoprotein 3, hepatocellular carcinoma, migration, miR-3150b, proliferation

## Abstract

**Background:**

In this study, we aimed to explore the potential involvement of miR-3150b in hepatocellular carcinoma (HCC) carcinogenesis.

**Methods:**

The expression of miR-3150b and Golgi phosphoprotein 3 (GOLPH3) was determined in HCC cell lines. Cell proliferation, migration and invasion were estimated by Cell Counting Kit-8, wound healing and Transwell assays. The association between miR-3150b and GOLPH3 was verified by luciferase assay.

**Results:**

MiR-3150b was downregulated, while GOLPH3 was remarkably upregulated in HCC cells. Furthermore, miR-3150b inhibited HCC cell proliferation, migration and invasion. MiR-3150b directly targeted and negatively regulated GOLPH3.

**Conclusion:**

MiR-3150b suppressed HCC cell proliferation, invasion and migration by targeting GOLPH3.

**Significance of this study**

**What is already known about this subject?**

- MiR-3150b has identified to be downregulated in several cancers.
- Through bioinformatics analysis, we identified miR-3150b might target Golgi phosphoprotein 3 (GOLPH3).
- GOLPH3 is a novel oncogene in hepatocellular carcinoma (HCC).

**What are the new findings?**

- MiR-3150b was downregulated in HCC cells.
- MiR-3150b inhibited HCC cell proliferation, migration and invasion.
- MiR-3150b inhibited HCC by targeting GOLPH3.

**How might these results change the focus of research or clinical practice?**

- MiR-3150b might be a valuable target for developing therapeutic strategy against HCC.

## Introduction

Hepatocellular carcinoma (HCC) ranks the third most common leading cause of cancer mortality, accounting for >500 000 deaths annually.^1 2^ Curative treatment options including surgical resection and liver transplantation lead to dissatisfactory overall survival.^3 4^ Although several patients with HCC have a response to interventional therapy, relapse represents the leading cause of treatment failure.^5 6^ Therefore, it is urgent to investigate the potential mechanism of HCC progression.

It has been shown that miRNAs, a crucial class of non-coding small RNAs of endogenous 21–23 nucleotides,^7 8^ are involved in regulating cell proliferation, metastasis, apoptosis and angiogenesis.^9 10^ Several lines of evidence suggest that the abnormal expression of miRNAs is associated with the carcinogenesis. Among them, miR-3150b has identified to be downregulated in several cancers, inducing laryngeal cancer,^11^ colorectal cancer^12 13^ and non-small cell lung cancer.^14^ However, the underlying role of miRNA-3150b on the HCC cell proliferation, invasion and migration is poorly understood.

Golgi phosphoprotein-3 (GOLPH3) is a well-known member of the trans-Golgi matrix family. Indeed, it has recently been shown that GOLPH3 functions as an oncogene in diverse tumors, including HCC.^15 16^ For example, Hu *et al* for the first time revealed that high GOLPH3 expression served as an independent predicator of poor prognosis in patients with HCC.^17^ Moreover, Li *et al* demonstrated that GOLPH3 downregulation suppressed HCC cell proliferation, migration, invasion in vitro.^18^ Additionally, previous studies have demonstrated that GOLPH3 induced tumorigenesis through activation of AKT signaling pathway accompanied by the phosphorylation of forkhead box protein O1, a vital transcriptional factor that regulated multiple cellular functions, including apoptosis, DNA damage repair and carcinogenesis.^19^

In the current study, we determined the expression of miRNA-3150b in HCC cells, and further investigated the effect of miRNA-3150b in HCC.

## Materials and methods

### Cell culture

Four human HCC cell lines and normal liver HL7702 cells were cultured in Dulbecco’s Modified Eagle Medium (Life Technologies, Carlsbad, California, USA) supplemented with 10% fetal bovine serum (FBS, Gibco, Carlsbad) with 5% CO_2_ at 37°C.

### Cell transfection

Using Lipofectamine 2000 (Invitrogen, Carlsbad), HepG2 and SNU-398 cells were transfected with inhibitor negative control (inhibitor NC), miR-3150b inhibitor, mimic negative control (mimic NC), miR-3150b mimic, si-GOLPH3 or together with pcDNA3.1-GOLPH3.

### Luciferase reporter assay

HEK-293T cells were co-transfected with miR-3150b mimic or mimic NC and the Luciferase miRNA Expression Reporter (Promega, Madison, Wisconsin, USA) comprising 3ʹ untranslated region (UTR) of GOLPH3 (wild or mutant type). At 48 hours post-transfection, the relative luciferase activity was calculated.

### Cell Counting Kit-8 assay

Cell proliferative ability was assessed using the Cell Counting Kit-8 (CCK-8; Beyotime, Shanghai, China) according to the manufacturer’s instructions. At 24, 48, 72, 96 hours post-transfection, cell viability was analyzed by a microplate reader (Bio-Rad Laboratories, Hercules, California, USA) at a wave length of 490 nm.

### RNA isolation and quantitative real-time PCR

Total RNA was isolated from cells using Trizol reagent (Invitrogen) based on the manufacturer’s instructions. Real-time PCR was subsequently carried out on the ABI 7500 Fast Real-Time PCR system (Applied Biosystems, Foster City, California, USA) using the SYBR Green PCR kit (Takara, Dalian, China). The relative expression levels of miR-3150b and GOLPH3 were normalized to U6 and GAPDH, respectively.

### Migration assay

After 0 or 24 hours transfection, scratch was performed using a 10 µL sterile pipette tip. Images were captured under a microscope (Leica Microsystems, Wetzlar, Germany) after incubation for 0 and 24 hours at 37 ºC.

### Transwell assay

Transwell assay was performed to assess the cell invasion using Transwell chambers (BD Biosciences, San Diego, California, USA). After 48 hours transfection, HepG2 and SNU-398 cells were plated in the upper chamber coated with matrigel matrix at a density of 5×10^5^ cells/mL. The lower chamber contained RPMI-1640 medium supplemented with 10% FBS. After stained with 4% crystal violet, cells were visualized under a microscope (×400 magnification).

### Western blot analysis

Protein lysate (30 µg) were separated by 10% sodium dodecyl sulfate-polyacrylamide gel electrophoresis and transferred onto polyvinylidene fluoride membranes, and blocked in 5% skimmed milk. The blots were incubated with rabbit antihuman polyclonal antibodies against GOLPH3 (catalog no. 19 112–1-AP; 1:2000 dilution; Peprotech, Rocky Hill, New Jersey, USA). In addition, GAPDH was used as an endogenous reference.

### Statistical analysis

Data were expressed as mean±SD. Statistical analysis was performed using analysis of variance with SPSS V.22.0 software (IBM, Armonk, New York, USA). The p value <0.05 was considered as statistically significant. Each experiment was conducted in triplicate.

## Results

### MiR-3150b expression was significantly downregulated and GOLPH3 was upregulated in HCC cell lines

**Figure 1:**
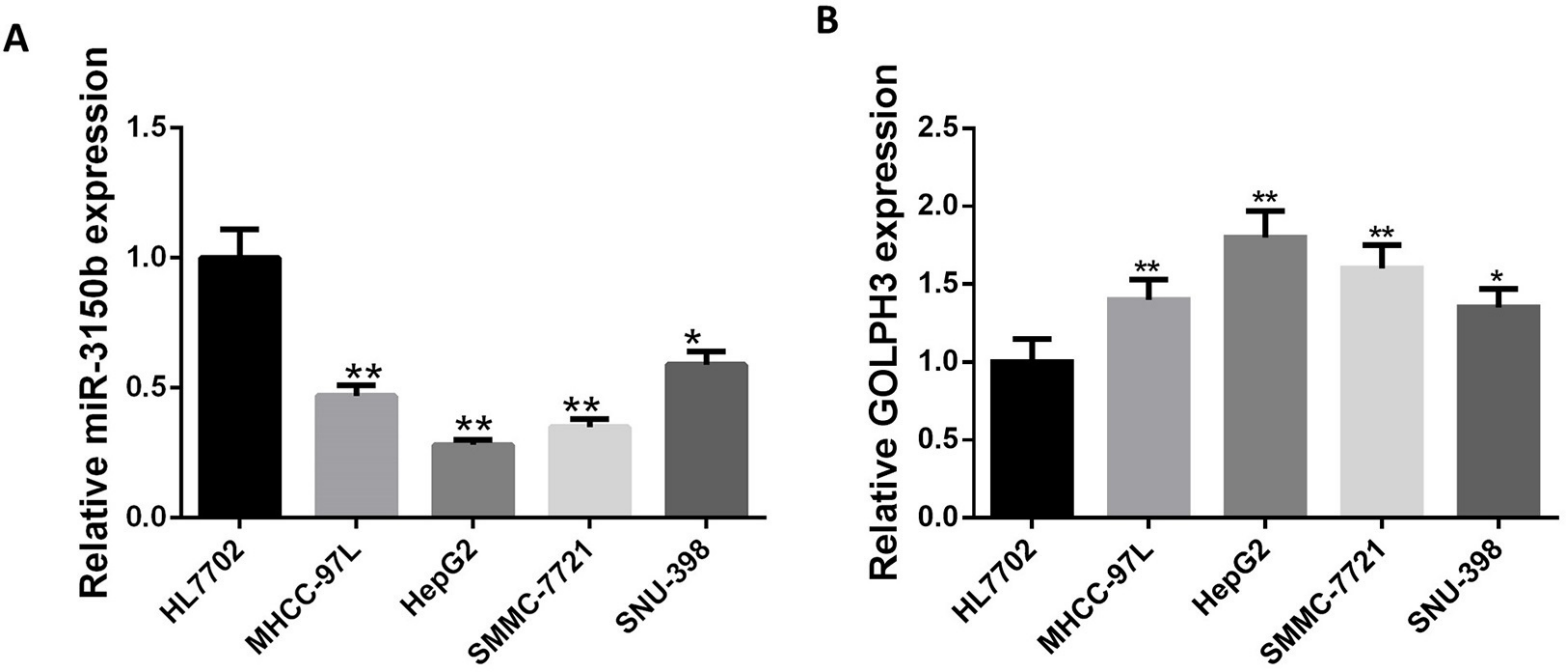
Expression of miR-3150b was downregulated in HCC cell lines. Relative mRNA expression of miR-3150b normal liver HL7702 cells and four GC cell lines with different differentiation status (MHCC-97L, HepG2, SMMC-7721 and SNU-398) determined by quantitative RT-PCR. All experiments were performed in triplicate with at least three independent experiments. Data were presented as the mean±SD. ^*^p<0.05, ^**^p<0.01 vs HL7702 cells. GOLPH3, Golgi phosphoprotein 3; HCC, hepatocellular carcinoma.

The relative mRNA expression of miR-3150b and GOLPH3 in four HCC cell lines with different differentiation status was first evaluated by qRT-PCR. MiR-3150b expression was significantly decreased in HCC cells compared with HL7702 cells (figure 1A), while GOLPH3 was highly expressed in HCC cells (figure 1B). Since the differential expression of miR-3150b and GOLPH3 was observed in HepG2 and SNU-398 cells, respectively, these two cell lines were chosen for the following experiments.

### MiR-3150b suppressed HCC cell proliferation

**Figure 2:**
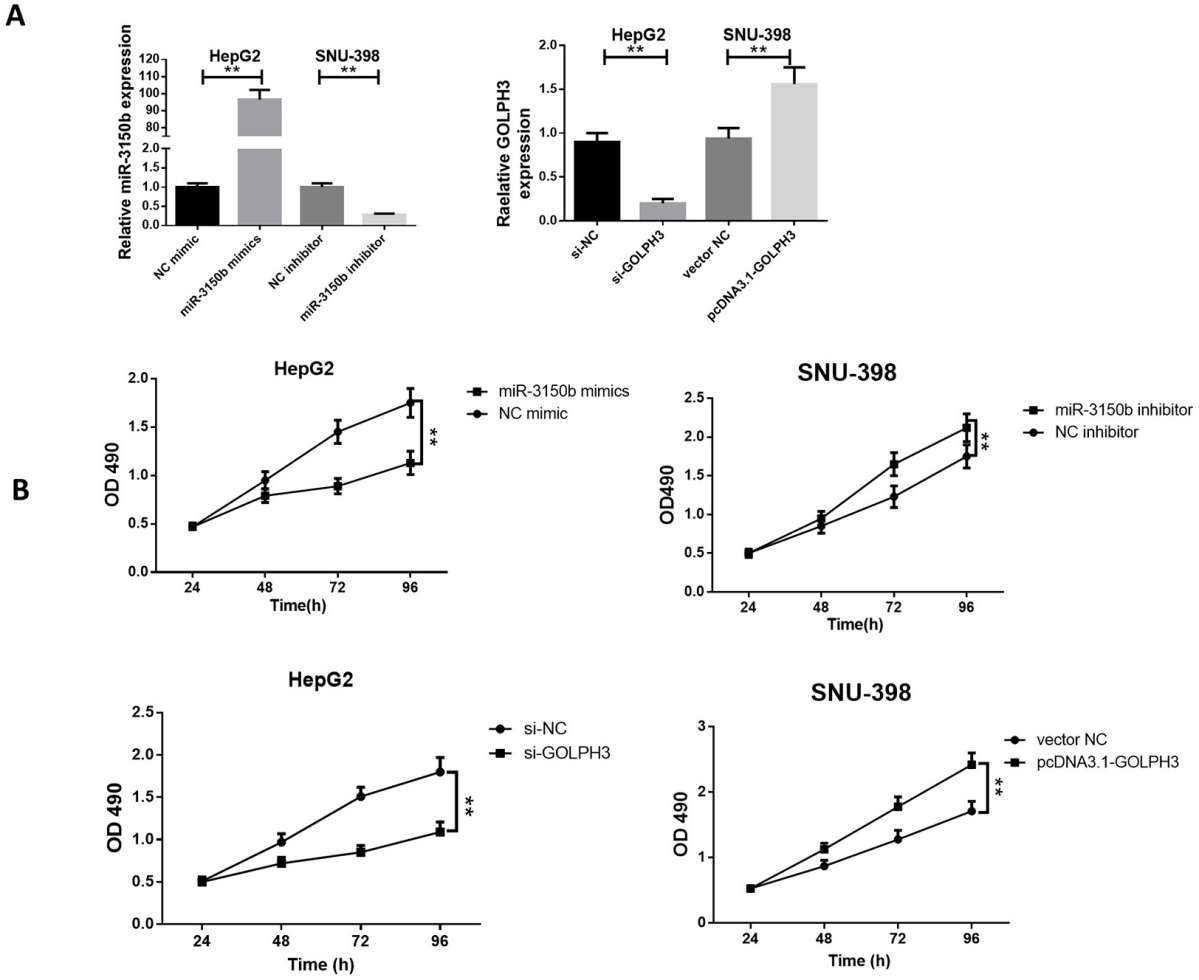
MiR-3150b suppressed HCC cell proliferation relative miR-3150b expression (A) and CCK-8 cell viability assay (B) in HepG2 cells with transfection of miR-3150b mimic or si-GOLPH3, and in SNU-398 cells transfected with miR-3150b inhibitor or pcDNA3.1-GOLPH3. All experiments were performed in triplicate with at least three independent experiments. Data were presented as the mean±SD. ^**^p<0.01 vs mimic NC or inhibitor NC group. CCK-8, Cell Counting Kit-8; GOLPH3, Golgi phosphoprotein 3; HCC, hepatocellular carcinoma; NC, negative control.

To investigate the underlying biological function of miR-3150b in HCC tumorigenesis, ectopic overexpression of miR-3150b was achieved by transfection of miR-3150b mimic into HepG2 cells, while miR-3150b knockdown was achieved by transfection of miR-3150b inhibitor into SNU-39 cells (figure 2A). The cell viability of HepG2 and SNU-398 cells at 24, 48, 72, 96 hours post-transfection was measured by CCK-8 assay. Overexpression of miR-3150b inhibited the proliferation of HepG2 cells, whereas miR-3150b knockdown enhanced the viability of SNU-398 cells. Interestingly, the inhibition of GOLPH3 by si-GOLPH3 or overexpression of GOLPH3 by pcDNA3.1-GOLPH3 led to similar results to miR-3150b overexpression or miR-3150b inhibition, respectively (figure 2B).

### MiR-3150b inhibited HCC cell migration and invasion

**Figure 3:**
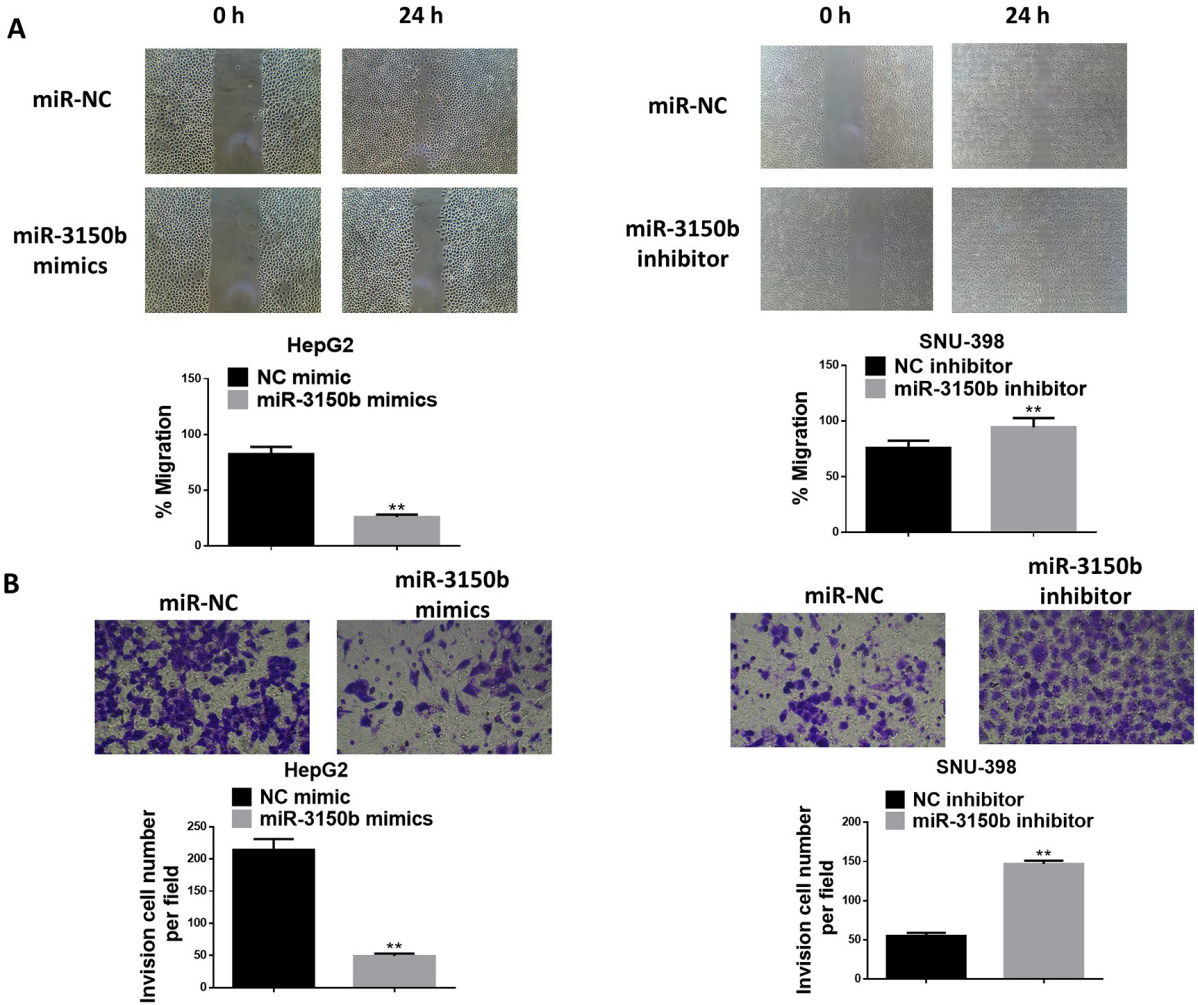
MiR-3150b suppressed HCC cell migration and invasion detection of HepG2 and SNU-398 cell migration (A) and invasion (B) by wound healing assay and Transwell assay. All experiments were performed in triplicate with at least three independent experiments. Data were presented as the mean±SD. ^**^p<0.01 vs mimic NC or inhibitor NC group. HCC, hepatocellular carcinoma; NC, negative control.

As shown in figure 3A, miR-3150b overexpresion observably decreased the migratory ability of HepG2 cells, whereas the migratory capacity of SNU-398 cells was markedly enhanced in response to treatment with miR-3150b inhibitor. MiR-3150b overexpression suppressed the invasion ability of HepG2 cells, while miR-3150b silencing augmented the invasion of SNU-398 cells (figure 3B).

### MiR-3150b directly targeted GOLPH3

**Figure 4:**
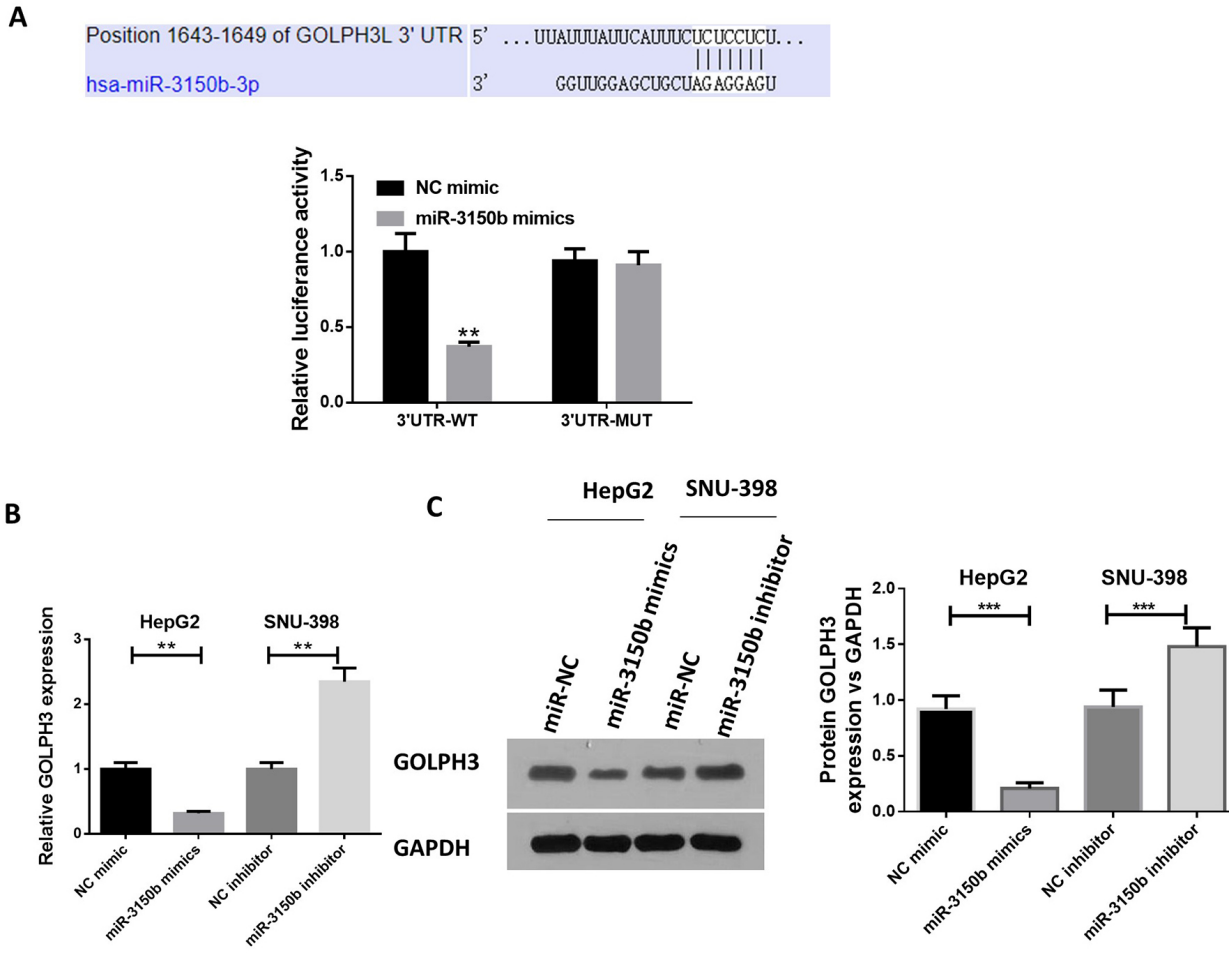
GOLPH3 was a direct target of miR-3150b. (A) alignment of miR-3150b with GOLPH3 3'-UTR sequences. HEK293T cells were co-transfected with luciferase reporter with wild-type (WT) GOLPH3 3'-UTR or with mutant GOLPH3 3'-UTR, and miR-3150b or mimic NC for 48 hours. The relative luciferase activity was analyzed by luciferase assay. The mRNA (B) and protein (C) levels of GOLPH3 in HepG2 cells transfected with miR-3150b mimic or mimic NC, and SNU-398 cells transfected with miR-3150b inhibitor or inhibitor NC using quantitative RT-PCR and western blot analysis. All experiments were performed in triplicate with at least three independent experiments. Data were presented as the mean±SD. ^**^p<0.01, ^***^p<0.001 vs mimic NC or inhibitor NC group. GOLPH3, Golgi phosphoprotein 3; HCC, hepatocellular carcinoma; NC, negative control; UTR, untranslated region.

Using TargetScan software, we identified the putative miR-3150b recognition sequence on the 3’UTR of GOLPH3 (figure 4A). The luciferase activity of wt-GOLPH3 was significantly suppressed by miR-3150b. Additionally, miR-3150b mimic reduced the mRNA and protein levels of GOLPH3 (figure 4B and C) in HepG2 cells. Meanwhile, miR-3150b inhibitor led to an opposite effect (figure 4B and C) in SNU-398 cells.

## Discussion

Compelling evidence has delineated dysregulated miRNAs in malignancy tumors, including HCC. For instance, Chen *et al* reported that upregulated miR-221 was positively correlated with HCC tumor stage, tumor nodes and microvascular invasion.^20^ Ge *et al* uncovered that miR-377 was a potential molecular target in HCC therapy.^21^ Chang *et al* demonstrated that miR-487a expression was an independent risk factor for disease-free survival in patients with HCC, and promoted HCC cell proliferation and metastasis.^22^ Therefore, better understanding of miRNAs in the pathogenesis of cancers might supply valuable insight for the prognosis and treatment.

Intriguingly, miRNA profile studies suggest that miR-3150b, located on 8q22.1, serves as a novel oncogene.^11–14^ In this paper, our data demonstrated that miR-3150b showed lower expression levels in HCC cell lines, indicating that miR-3150b might act as an anti-oncogene in the development and progression of HCC. Therefore, we speculated that miR-3150b might negatively regulate tumor growth. The results of CCK-8 assay showed that miR-3150b knockdown enhanced HCC cell proliferation. Further functional analysis showed that miR-3150b mimic could inhibit the migration and invasion progression of HepG2 and SNU-398 cells, while depletion of miR-3150b led to an opposite effect.

GLOPH3 is a famous oncogene. For example, downregulation of GOLPH3 by RNA interference inhibited glioma cell migration and invasion via the mTOR-YB1 pathway.^23^ Recently, Dai *et al* found that GOLPH3 overexpression alleviated cisplatin-induced HCC cell apoptosis, and promoted the aggressiveness of HCC cells via NF-κB pathway.^16^ In the present study, GLOPH3 was confirmed to be a target of miR-3150b. Moreover, gain-of-function and loss-of-function assay showed that miR-3150b overexpression negatively regulated GOLPH3 expression in HCC cells, while miR-3150b knockdown led to an opposite effect. However, several limitations were included in our study. First, the in vivo experiments were excluded. Second, the underlying mechanisms for GOLPH3 in HCC development were not deeply studied. Third, whether miR-3150b has some other targets or not is still unclear. Lastly, the in vivo effects of miR-3150b in HCC still need to be confirmed.

To conclude, our study provided the first demonstration that miR-3150b exerted a tumor-suppressive role in HCC, at least in part, by targeting GOLPH3.
